# Association of BMI category with change in children’s physical activity between ages 6 and 11 years: a longitudinal study

**DOI:** 10.1038/s41366-019-0459-0

**Published:** 2019-11-12

**Authors:** Russell Jago, Ruth Salway, Lydia Emm-Collison, Simon J. Sebire, Janice L. Thompson, Deborah A. Lawlor

**Affiliations:** 1 Centre for Exercise, Nutrition & Health Sciences, School for Policy Studies, University of Bristol, 8 Priory Road, Bristol, BS8 1TZ UK; 2 School of Sport, Exercise and Rehabilitation Sciences, University of Birmingham, Birmingham, B15 2TT UK; 3 MRC Integrative Epidemiology Unit, University of Bristol, Oakfield House, Oakfield Grove, Bristol, BS8 2BN UK; 4 Population Health Sciences, Bristol Medical School, University of Bristol, Canynge Hall, Whiteladies Road, Bristol, BS8 2PS UK

**Keywords:** Obesity, Cardiovascular diseases

## Abstract

**Background/objectives:**

To examine the association of body mass index (BMI) with change in children’s physical activity and sedentary time between ages 6 and 11.

**Participants:**

A total of 2132 children participated from 57 schools in Southwest England, from the B-PROACT1V study.

**Methods:**

Mean minutes of MVPA and sedentary time per day were derived from accelerometer-based measurements at ages 6, 9 and 11. Linear multilevel models examined the association of BMI categories with MVPA and sedentary time between 6 and 11, adjusting for seasonality, wear time, gender and household education. Differences in change over time were examined using interaction terms.

**Results:**

Average weekday MVPA decreased between ages 6 and 11 by 2.2 min/day/year (95% CI: 1.9 to 2.5), with a steeper decline at weekends. Average sedentary time increased at a rate of 12.9 min/day/year (95% CI: 12.2 to 13.6). There were no differences in mean levels of MVPA by BMI categories at age 6, but differences emerged as children aged, with the gap between children who were healthy weight and overweight increasing by 1.7 min/day (95% CI: 0.8–2.6) every year, and between healthy and obese by 2.0 min/day (95% CI: 0.9–3.1) each year. Children who were overweight/obese engaged in less average weekday sedentary time at age 6 than those of healthy weight, but the gap closed by age 11.

**Conclusion:**

MVPA declines and sedentary time increases on average for all children between ages 6 and 11. While there are no differences in activity levels by BMI category at age 6, differences in MVPA emerge over time for those who are overweight and obese. Developing interventions that support children to retain activity levels as they approach older childhood, particularly those who are overweight/obese could improve public health.

## Introduction

Physical activity is associated with improved psychological well-being and lower levels of cardiometabolic risk factors among children and adolescents [1, 2]. There is evidence that lower amounts of time spent being sedentary are also associated with improved physical and psychological health among children and adolescents, but it is unclear if these associations are independent of physical activity [3, 4]. Physical activity levels change with age, increasing between ages 3 and 6, and appearing to peak around the age that children start school (approximately age 6) while sedentary time is relatively stable in this age range [5]. However, several studies have shown that physical activity declines and sedentary time increases in an approximately linear fashion between ages 6 and 15, with girls less active than boys across all age groups [6, 7]. As a result, large proportions of older children are not engaging in the recommended 60 min per day of moderate-to-vigorous-intensity physical activity (MVPA) [6, 8, 9]. Examining patterns of physical activity across childhood is important for identifying key ages in which to intervene to change behaviour.

Data from the 2017/18 National Child Measurement Programme showed that 10% of 4–5-year olds in England were obese, increasing to 20% of 10–11-year olds [10]. Cross-sectional studies have found that children who are overweight or obese are less physically active than children who are a healthy weight after the age of 6 years [6, 11, 12]. It is not clear, however, if these differences are consistent through childhood or if the nature of the association changes as children age. This is important as accumulating evidence highlights the significance of early body mass index (BMI) on the later risk of obesity. For example, 90% of children who are obese at age three remain overweight or obese in adolescence [13]. As such, understanding the nature of any difference in physical activity by BMI group and the age at which any differences occur would inform the design of interventions.

Understanding the factors that may explain the age-related decline in children’s physical activity and whether the impact of those variables changes over time is important for guiding prevention efforts. Current data from large collaborations such as the International Children’s Accelerometer Database (ICAD) provide useful data on differences by age and geographical comparisons but lack information on within-person change [6]. Moreover, contemporary within-person studies are particularly important as several of the studies included in ICAD were conducted over 10 years ago and recent evidence suggests that there have been secular declines in MVPA over the past decade. For example, a Norwegian study has shown that 9-year-old girls engaged in an average of 4.2 fewer minutes of MVPA per day in 2011–2012 than in 2004–2005 [14]. Fewer studies have looked at longitudinal change. In a previous paper using data from the first two data collections within the B-PROACT1V study, we showed that time spent in MVPA reduced from 72 to 69 min per day between ages 6 and 9 for boys while girls reduced from 62 to 56 min per day, with increases in sedentary time for both boys and girls [8]. This provides key indications of the change in activity levels between ages 6 and 11, including possible differences between girls and boys, but is limited to two time points and does not include data on the end of primary school at age 11, the age when the majority of other studies in the field have been conducted [6]. The Gateshead Millennium Study found that MVPA declined between the ages of 8 and 15, but there was no evidence that the decline either began or increased at adolescence [15]. Further analysis found that adiposity influenced physical activity but not vice versa in the same cohort [16]. The study did not, however, look at how physical activity differs by subsequent BMI classification. Understanding how associations may differ by BMI classification group as children age is essential for understanding where to prioritise prevention efforts. For example, if there is a clear association between BMI classification and physical activity among children who are obese at a young age it may be most prudent to use limited funds to maintain and increase the physical activity of this group.

Examining how BMI is associated with change in activity levels over time is complex, as confounders such as gender and socio-economic indicators may also be associated with change over time. For example, studies consistently show that girls are less active and more sedentary than boys at all ages, but it is not known whether this gap widens over time. The prevalence of obesity among 11-year-old children living in the most deprived areas in England is more than double that in the least deprived areas (27% vs. 12%) [10] and data from the ALSPAC longitudinal study showed that socio-economic differences in childhood BMI began to emerge at 4 years, becoming stronger with age with marked differences between parental education groups by age 7 [17]. Thus, a child’s BMI, gender and socio-economic position may all impact on the amount of physical activity in which a child engages and if their engagement changes over time. To identify how to optimise new prevention efforts, there is a need to examine how BMI is associated with physical activity in childhood, and if associations change as children age. The aim of this paper was to examine the association of BMI with change in MVPA and sedentary time between ages 6 and 11, accounting for differences in gender and household education.

## Methods

### Design and participants

**Fig. 1:**
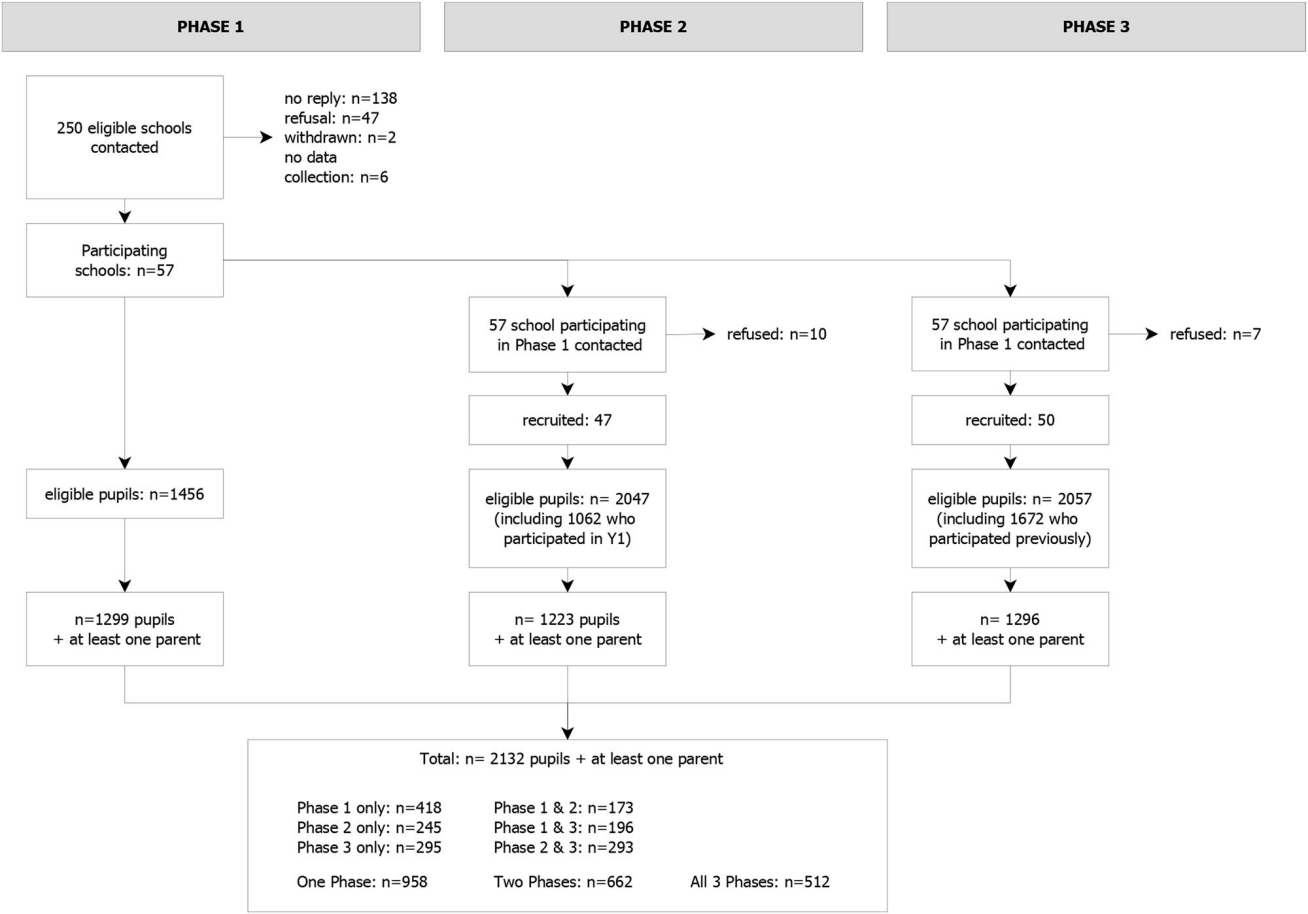
Flow diagram of recruitment for Phases 1–3 of the B-PROACT1V study

B-PROACT1V is a longitudinal study that aimed to examine the physical activity and sedentary behaviours of primary school children aged 5–11 years, and their parents [8, 18]. The study received ethical approval from the School of Policy Studies Ethics Committee at the University of Bristol, UK, and written parental consent was received for all participants [19]. In Phase 1 (data collection between January 2012 and July 2013), all children in Year 1 of primary school (aged 5–6 years) from 57 schools in and around Bristol were invited to participate. In Phases 2 and 3, when the children were in Year 4 (aged 8–9, data collection between March 2015 and July 2016) and Year 6 (aged 10–11; data collection between March 2017 and May 2018), all schools from Phase 1 were invited to participate, and all children within participating schools were eligible in Phases 2 and 3, regardless of whether they had participated in Phase 1. A total of 47 schools participated in Phase 2 and 50 schools in Phase 3. Across the three time points, data were collected for 2132 children with 1299 children in Year 1 (age 6), 1 223 children in Year 4 (age 9), and 1296 in Year 6 (age 11). There were 1174 children who provided data for at least two phases, and 512 children included in all three phases (Fig. 1).

### Measurements

#### Accelerometer data

Children wore a waist-worn ActiGraph wGT3X-BT accelerometer for 5 days, including two weekend days. Accelerometer data were processed using Kinesoft (v3.3.75; Kinesoft, Saskatchewan, Canada) and analysis was restricted to those children who provided at least two days of valid weekday data and one valid weekend day to provide a compromise that captures a typical day but maximises the sample size to enable separate analysis of weekdays and weekends. A valid day was defined as at least 500 min of data, after excluding intervals of ≥60min of zero counts allowing up to two minutes of interruptions [6]. Data were recorded at 10 s intervals and characterised as sedentary, light or MVPA using Evenson population-specific cut points for children [20]. The average number of MVPA and sedentary minutes per weekday and weekend day were derived for each child. In addition, we recorded the average number of minutes the accelerometer was worn on weekdays and weekends.

#### Height, weight and BMI

Child height and weight were recorded to the nearest 0.1 cm and 0.1 kg, respectively, by trained fieldworkers at each time point. Parents were asked ‘What is your child’s gender?’ Age was calculated from parent-recorded date of birth, which was cross-referenced across the three phases, and any child with missing age data (*n* = 280 (13%)) was assigned the average age for the school in the data collection period. BMI was calculated and converted to an age- and sex-specific standard deviation score based on UK reference curves [21, 22]. These were used to create age- and sex-specific overweight and obesity indicators using 85th and 95th percentiles, respectively. As less than 2% of children were underweight, we did not analyse them as a separate category but included them in the healthy weight category.

#### Household education

At Phase 1, an adult in the household was asked, via a questionnaire, their highest educational qualification, while at Phases 2 and 3, they were asked the highest education qualification of anyone in the household. Due to this change, it was not possible to construct a baseline measure of education at age 6, and so instead we combined these to identify the highest household educational qualification recorded at any phase. The categories were ‘Up to GCSE/O level or equivalent’ (UK qualification usually acquired at age 16), ‘A level/NVQ or equivalent’ (qualification usually acquired at age 18), ‘University Degree/HND or equivalent’ and ‘Higher Degree (MSc/PhD) or equivalent’.

### Statistical analysis

There were 1924 participants (90%) with valid weekday data (and 1773 (83%) for weekend data) for at least one time point, 970 (46%; 839 (39%) weekend) for at least two time points and 381 (18%; 286 (13%) weekend) at all three time points. We reported descriptive summaries of key variables over time, and examined the movement between healthy weight, overweight and obese categories at different time points.

Linear multilevel models, with random effects at the school and child level, to account for clustering and repeated measures respectively, were used to determine mean levels of physical activity at age 6 and the change in physical activity between ages 6 and 11. As we only had data at three distinct ages (with no measurements in between these ages) we had to assume a linear change in activity over time between ages 6 and 11. We used restricted maximum likelihood for more accurate estimation of standard errors. While multiple imputation can be used to deal with missing data, it is not straightforward for multilevel models as it is not easy to account for the complex model structure. Thus, we restricted the analysis to complete cases only. However, multilevel models maximise the use of data as they include all participants who have data for at least one measure, under a missing at random assumption. Models were adjusted for accelerometer wear time as this differed across children, with age and time of year of data collection. As data collection occurred in different schools at different times of the year, and MVPA varies across the year [23], we adjusted for seasonal variability using harmonic sine and cosine functions [24]. This models the seasonal trend as a function that varies smoothly from one month to the next.

We analysed MVPA and sedentary time on weekdays and weekend days separately, as previous research [25, 26] suggested that levels and patterns of physical activity may differ between weekdays and weekend. We explored four models. Model 1 regressed age on the outcome (MVPA and sedentary time), controlling adjusting for seasonality and wear time. Models 2 and 3 additionally included gender and household education respectively as main and age-interaction terms. Finally, Model 4 was based on Model 1 and included BMI category from the three different time points, adjusting for gender and household education as confounders, and including interactions of BMI category, gender and household education with age. As children change BMI categories over the study duration, we used BMI category measured the week before the corresponding physical activity measure. This captures the association between current BMI and physical activity rather than baseline BMI at age 6, and the resulting models estimate trajectories of change over time for the nonvarying confounders gender and household education, with additional estimates associated with a child being overweight/obese at any given age. In addition, we re-ran all models using three valid weekdays of data and two valid weekends, in a sensitivity analysis. All analyses were performed in Stata version 15.0 (Statcorp, College Station, TX).

## Results

Missing data within phases ranged from 1 to 25% (Table 1). The summary characteristics (Table 1) showed a decrease in average MVPA between ages 6 and 11 on both weekdays and weekends, and an increase in sedentary time. Over the same period, the average BMI *z*-score increased. The percentage who were overweight increased from 11% at age 6 to 14% at age 11, while the percentage who were obese increased from 8 to 15%. Children who were overweight at one time point were more likely to remain overweight or become obese than to become healthy weight, and those who were obese typically remained obese at the next time point (Table S1).

**Table 1:** Comparison of characteristics and % of missing data across time points

	Age 6		Age 9		Age 11	
	*N*	% or mean (sd)	*N*	% or mean (sd)	*N*	% or mean (sd)
*N*	1299		1223		1296	
BMI *z*-score	1271	0.27 (0.95)	1217	0.35 (1.07)	1285	0.35 (1.16)
% Normal weight		81%		75%		71%
% Overweight		11%		11%		14%
% Obese		8%		13%		15%
% female	1299	49%	1223	55%	1296	52%
Highest household education	1176		1125		1191	
Up to GCSE or equiv		20%		19%		20%
A level or equiv		28%		29%		26%
University degree or equiv		36%		36%		37%
Higher degree		16%		17%		17%
Average weekday activity						
MVPA (min)	1087	68.0 (21.1)	1059	62.9 (22.0)	1129	60.6 (23.1)
Light (min)	1087	244.2 (39.9)	1059	223.6 (41.7)	1129	210.1 (42.7)
Sedentary time (min)	1087	372.0 (59.3)	1059	445.7 (64.5)	1129	480.4 (69.8)
Wear time (min)	1087	684.2 (68.4)	1059	732.2 (68.5)	1129	751.1 (76.6)
% of time in MVPA	1087	10%	1059	9%	1129	8%
% of time in sedentary	1087	54%	1059	61%	1129	64%
Average weekend activity						
MVPA (min)	980	66.3 (27.6)	942	61.5 (31.8)	976	53.4 (31.3)
Light (min)	980	234.0 (46.6)	942	215.1 (50.2)	976	195.2 (49.8)
Sedentary time (min)	980	339.1 (76.0)	942	401.9 (83.7)	976	441.8 (88.8)
Wear time (min)	980	639.5 (85.2)	942	678.5 (90.3)	976	690.4 (95.9)
% of time in MVPA	980	10%	942	9%	976	8%
% of time in sedentary	980	53%	942	59%	976	64%
% MVPA > 60 min	1023	63%	1026	46%	1062	41%

In analyses adjusted for wear time and seasonality, weekday MVPA decreased between ages 6 and 11 by 2.2 min/day/year (95% CI: 1.9–2.5), with a steeper decline at weekends of 3.1 min/day/year (95% CI: 2.6–3.6) (Table S2). There were differences between girls and boys both in the mean MVPA levels at age 6, with girls less active than boys, and in the decline over time which was steeper for girls than for boys. Thus, the gap between boys and girls increased by around 50% from 9.9 min at age 6–14.8 min at age 11. Girls of healthy weight engaged in less MVPA than boys with obesity at all time points. Household education was associated with weekday MVPA, with children whose parents are educated to a higher level less active at age 6. There were weak associations with change in weekday MVPA, with the decline in MVPA slightly less steep for those children in higher educated households. There was no association between household education and weekend MVPA.

Weekday sedentary time increased at a rate of 12.9 min/day/year (95% CI: 12.2–13.6) with similar rates at weekends of 13.9 min/day/year (95% CI: 13.0–14.9) (Table S3). Girls were more sedentary at age 6 than boys, but their sedentary time increased at a similar rate over time. Household education was associated with sedentary time on weekdays at age 6, and weakly associated with the change in weekday sedentary time between ages 6 and 11, but not on weekend days.

**Fig. 2:**
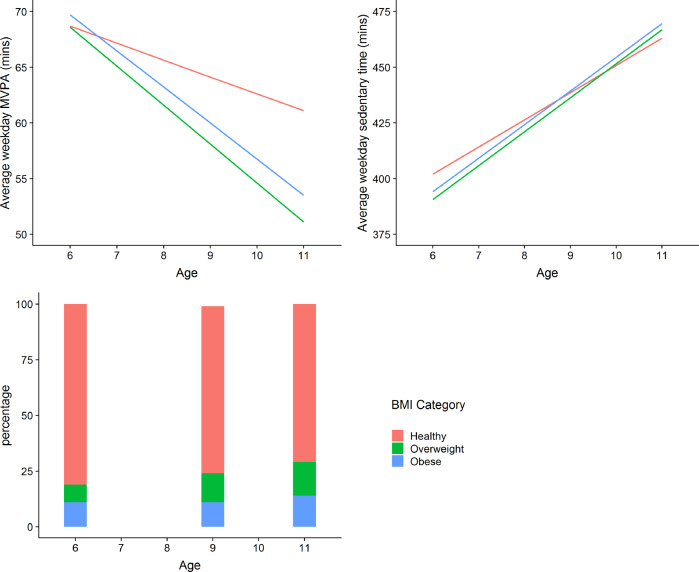
Change in MVPA (top left), sedentary time (top right) by BMI category and change in BMI category (bottom) over time

Table 2 reports the associations between current BMI category and MVPA, adjusting for seasonality and wear time and additionally for the confounders gender and household education (full details in Table S4), and shows the differences in mean MVPA and change in MVPA by year for different BMI categories compared to a similar child (i.e. same gender and household education) who is of healthy weight. Figure 2 shows the weekday MVPA and sedentary time between ages 6 and 11 for different BMI categories, averaging over gender and household education; the changing proportions of children who were healthy, overweight and obese are shown for comparison. At age 6, there were no differences between BMI categories, with similar mean daily MVPA levels for those who were healthy weight, overweight and obese, when adjusting for differences in gender and household education. However, BMI category at subsequent ages was associated with MVPA on weekdays but not weekends, with lower MVPA for children who were overweight and obese compared to those of healthy weight. Thus, a child who is obese at age 6, and remains so at age 11, engaged in 10.0 min less MVPA per weekday (95% CI: 6.9–13.0 min) at age 11 than a similar child of healthy weight at both ages. Patterns were different for weekday sedentary time (Tables 3 and S5), with children who were overweight or obese engaging in less average sedentary time at age 6 than those of healthy weight. As children aged, the gap between those who were healthy weight and those overweight or obese narrowed. So at age 6, a child who is obese engaged in 11.3 min less sedentary time (95% CI: 1.8–21.0 min) than a similar child of healthy weight, but by age 11, if both children remained obese and heathy weight respectively, the gap disappeared, with the child who was obese engaging in 3.8 min more sedentary time (95% CI: −3.0 to 10.7). There were no associations between BMI category and weekend sedentary time. We re-ran all models using three valid weekdays of data and two valid weekends, in a sensitivity analysis (Table S6) but the magnitude and direction of estimates were the same, and the results did not change.

**Table 2:** Change in MVPA over time by BMI category: difference from healthy weight

	Weekday			Weekend		
	Est	95% CI	*p*-value	Est	95% CI	*p*-value
Model estimates	*n* = 1742			*n* = 1624		
Mean MVPA at age 6 (min/day)						
BMI category						
Healthy	0	Reference		0	Reference	
Overweight	0.99	(−2.41, 4.38)		−0.95	(−6.25, 4.35)	
Obese	−0.03	(−4.26, 4.21)	0.846	−6.82	(−13.66, 0.03)	0.147
Change in MVPA (min/day/year)						
BMI category						
Healthy	0	Reference		0	Reference	
Overweight	−1.71	(−2.65, −0.78)		−1.31	(−2.79, 0.17)	
Obese	−1.99	(−3.05, −0.92)	< 0.0005	−0.65	(−2.42, 1.12)	0.195
Comparisons with similar child of health weight^a^						
Overweight						
Age 6	0.99	(−2.41, 4.38)		−0.95	(−6.25, 4.35)	
Age 9	−4.15	(−6.26, −2.03)		−4.88	(−8.17, −1.60)	
Age 11	−7.58	(−10.49, −4.66)		−7.51	(−12.12, −2.89)	
Obese						
Age 6	−0.03	(−4.26, 4.21)		−6.82	(−13.66, 0.03)	
Age 9	−5.98	(−8.40, −3.56)		−8.77	(−12.53, −5.00)	
Age 11	−9.96	(−12.98, −6.93)		−10.06	(−14.92, −5.21)	

*p*-values: Test for differences between BMI categories

Model adjusted for seasonality, wear time, gender, household education and age-interactions with gender and household education

^a^The difference in average MVPA between a child of healthy weight at baseline who remains so, and a child who is overweight/obese at baseline and remains so, at each age

**Table 3:** Change in sedentary time over time by BMI category: difference from healthy weight

	Weekday			Weekend		
	Est	95% CI	*p*-value	Est	95% CI	*p*-value
Model estimates	*n* = 1742			*n* = 1624		
Mean sed time at age 6 (min/day)						
BMI category						
Healthy	0	Reference		0	Reference	
Overweight	−7.90	(−15.57, −0.22)		−3.28	(−13.88, 7.31)	
Obese	−11.41	(−21.00, −1.82)	0.014	−4.06	(−17.79, 9.68)	0.728
Change in sed time (min/day/year)						
BMI category						
Healthy	0	Reference		0	Reference	
Overweight	2.90	(0.80, 5.01)		1.91	(−1.04, 4.86)	
Obese	3.05	(0.65, 5.45)	0.002	1.50	(−2.01, 5.01)	0.352
Comparisons with similar child of health weight^a^						
Overweight						
Age 6	−7.90	(−15.57, −0.22)		−3.28	(−13.88, 7.31)	
Age 9	0.81	(−3.98, 5.61)		2.44	(−4.16, 9.04)	
Age 11	6.61	(0.02, 13.21)		6.26	(−2.96, 15.47)	
Obese						
Age 6	−11.41	(−21.00, −1.82)		−4.06	(−17.79, 9.68)	
Age 9	−2.26	(−7.79, 3.27)		0.44	(−7.23, 8.11)	
Age 11	3.84	(−3.03, 10.72)		3.44	(−6.33, 13.20)	

*p*-values: Test for differences between BMI categories

Model adjusted for seasonality, wear time, gender, household education and age-interactions with gender and household education

^a^The difference in average sedentary time between a child of healthy weight at baseline who remains so, and a child who is overweight/obese at baseline and remains so, at each age

## Discussion

The data presented in this paper have highlighted that on average, time spent in MVPA decreases and sedentary time increases in children between ages 6 and 11, amounting to 10.9 min/day less average weekday MVPA (15.5 min/day at weekends) and 64.5 min/day more average weekday sedentary time (68.6 min/day at weekends) over the five years. At age 6, there were no differences in the average MVPA levels of children who were healthy weight, overweight and obese. However, current BMI category was associated with weekday MVPA as children aged, with lower weekday MVPA for children who were overweight and obese, and this association became stronger with age. So, a child of healthy weight at age 11 (71% of children) engaged in an average of 6.6 min/day less MVPA than a similar child of healthy weight at age 6, while a child who was overweight (14% of children) engaged in 15.1 min/day less at age 11 than a similar child who was overweight at age 6, and a child with obesity (15% of children) engaged in 16.5 min/day less. Moreover, the proportion of children who are overweight or obese increased between age 6 and 11, with the majority (78%) at age 6 remaining so by age 11 and some of those who were a healthy weight at age 6 becoming overweight/obese. A key implication of these findings is that the differences in MVPA between BMI categories increase over time, becoming more marked as the children age. The findings therefore suggest a need for dedicated work on the physical activity patterns of children who are overweight or obese at the start of primary school. Research is needed to identify ways to help this group of children remain active and attenuate both the age-related decline in physical activity and the additional decrease associated with being overweight or obese when compared to children who are healthy weight. As such, identifying the cause of the weekday differences and the patterns of behaviour on weekdays for the different groups is important for identifying potentially helpful behaviour change programmes.

These longitudinal findings complement the recent cross-sectional analysis of the ICAD database [6] which found that physical activity did not differ by BMI categories in younger children, but that being overweight/obese was associated with being less active from around 7 years of age. A number of studies have shown higher BMI among children with lower levels of activity [27–29]. The causal relationship between BMI and physical activity is complex, with a recent longitudinal cohort study [16] finding that adiposity influences activity, but not the other way round, and a Mendelian randomisation analysis of ALSPAC data [30] concluding that increased adiposity is a causal risk factor for lower physical activity in children. In our study, BMI measurements precede physical activity measurements and so our results therefore suggest that after age 6, children who are overweight/obese are less active, and that the gap between obese and healthy weight increases over time, which may in turn contribute to higher BMI. It would be interesting to explore causal associations more fully in future, although potentially difficult with these data because of the large gaps between measurements. Collectively, our data and previous work highlight how differences in physical activity in relation to changing BMI develops as children age and that there is a need to ensure that all groups of children are as active as possible so that there is less opportunity for differences in activity between groups to increase as children age. In addition, strategies that help younger children achieve a healthy weight before the gap appears may have implications for their long-term physical activity levels.

As has been seen elsewhere, we saw marked differences in physical activity levels between boys and girls, with girls engaging in less MVPA and more sedentary time on both weekdays and weekends than boys at age 6. Girls’ MVPA also declined at a faster rate, so that the gap between boys and girls increases between ages 6 and 11. We also saw differences by household education in weekday MVPA and sedentary time at age 6, with children in households with higher qualifications engaging in less MVPA and more sedentary time. However, children from lower-education households were more likely to become overweight or obese over the time period, and so by age 11, the impact of BMI overrides any association with household education. As a result, household education differences are no longer evident by age 11. This suggests that efforts to tackle differences in MVPA after the age of 6 should target gender and BMI rather than socio-economic groups.

### Strengths and limitations

The major strength of this study is the in-depth provision of accelerometer and BMI data at three time-points during childhood, which has facilitated the assessment of within-person change over time. This has allowed us to examine how the association between BMI classification group and physical activity and sedentary time changes during primary school, which as highlighted above, makes a unique and important contribution to the evidence base. The use of multilevel models maximises the data used by including data from all study participants. However, our study is limited as physical activity data were assessed at three distinct ages and so we had to assume a linear change in MVPA and sedentary time with age. It is possible that this is a misspecification, and large departures from linearity would mean our results are biased. Finally, data are from the area around a single UK city which was predominantly White British, which limits the ability to generalise to other settings, contexts and ethnic groups.

## Conclusions

MVPA declines and sedentary time increases on average for all children between ages 6 and 11. While there are no differences in activity levels by weight status at age 6, differences in MVPA emerge over time for those who are overweight and obese. Developing and testing tailored interventions that support children to maintain activity levels as they approach older childhood, particularly those who are overweight/obese could improve public health.

## Supplementary information

Supplementary Tables S1 to S6
