# Correction: Association of BMI category with change in children’s physical activity between ages 6 and 11 years: a longitudinal study

**DOI:** 10.1038/s41366-021-00827-0

**Published:** 2021-05-05

**Authors:** Russell Jago, Ruth Salway, Lydia Emm-Collison, Simon J. Sebire, Janice L. Thompson, Deborah A. Lawlor

**Affiliations:** 1 Centre for Exercise, Nutrition & Health Sciences, School for Policy Studies, University of Bristol, Bristol, UK; 2 School of Sport, Exercise and Rehabilitation Sciences, University of Birmingham, Birmingham, UK; 3 MRC Integrative Epidemiology Unit, University of Bristol, Bristol, UK; 4 Population Health Sciences, Bristol Medical School, University of Bristol, Bristol, UK

**Keywords:** Obesity, Cardiovascular diseases

Correction to: *International Journal of Obesity*

10.1038/s41366-019-0459-0

The original version of this article unfortunately contained a mistake. There was a typographical error in Table 2. The 7th row of the table should read “Change in MVPA (min/day/year)”. The authors apologize for the error. The original article has been corrected.

