# Supplementary material for: Association of BMI category with change in children’s physical activity between ages 6 and 11 years: a longitudinal study
Source: Int J Obes (Lond). 2019 Nov 12;44(1):104–13. doi: 10.1038/s41366-019-0459-0 (PMC6923172; doi:10.1038/s41366-019-0459-0)
Supplement: Supplementary file 1 — Supplementary Tables S1 to S6 [file 41366_2019_459_MOESM1_ESM.docx]

SUPPLEMENTARY MATERIAL

**Association of BMI category with change in children’s physical activity between ages 6 and 11 years: a longitudinal study**

Russell Jago, Ruth Salway, Lydia Emm-Collison, Simon J. Sebire, Janice L. Thompson and Deborah A. Lawlor

Table S1: Movement between healthy, overweight and obese categories

Table S2: Associations with change in MVPA over time: models for all data, by gender and by household education

Table S3: Associations with change in sedentary time over time: models for all data, by gender and by household education

Table S4: Associations of BMI category with change in MVPA over time: full model details

Table S5: Associations of BMI category with change in sedentary time over time: full model details

Table S6: Change in MVPA and sedentary time over time by BMI category for 3 valid weekdays and 2 valid weekends: sensitivity analysis

**Table S1: Movement between healthy, overweight and obese categories**

|  | % of age 6 category at age 9 | | |  |
| --- | --- | --- | --- | --- |
| Age 6 | Normal | Overweight | Obese | % at age 6 |
| Normal | 87% | 10% | 4% | 83% |
| Overweight | 28% | 32% | 39% | 11% |
| Obese | 3% | 3% | 95% | 6% |
| % at age 9 | 74% | 13% | 13% | 100% |

These are row percentages, so for example, of those who were overweight at age 6, 32% were still overweight at age 9.

|  | % of age 6 category at age 11 | | |  |
| --- | --- | --- | --- | --- |
| Age 9 | Normal | Overweight | Obese | % at age 9 |
| Normal | 89% | 10% | 1% | 76% |
| Overweight | 24% | 45% | 31% | 12% |
| Obese | 3% | 10% | 87% | 12% |
| % at age 11 | 70% | 14% | 16% | 100% |

These are row percentages, so for example, of those who were overweight at age 9, 45% were still overweight at age 11.

**Table S2: Associations with change in MVPA over time: models for all data, by gender and by household education**

|  |  | | Weekday | | |  | Weekend | | |
| --- | --- | --- | --- | --- | --- | --- | --- | --- | --- |
|  |  | | Est | 95% CI | p-value |  | Est | 95% CI | p-value |
| **Model 1** | | | **n=1924** | **n=1773** | | | |  | |
| Mean MVPA at age 6 (min/day) | | | 69.28 | (67.59, 70.98) |  |  | 68.52 | (66.38, 70.65) |  |
| Change in MVPA (min/day/year) | | | -2.17 | (-2.49, -1.85) | <0.0005 |  | -3.10 | (-3.58, -2.61) | <0.0005 |
| **Model 2** | | **n=1924** | | **n=1773** | | | |  | |
| **Mean MVPA at age 6 (min/day)** | | | 74.08 | (72.11, 76.05) |  |  | 72.08 | (69.45, 74.71) |  |
| Gender | | |  |  |  |  |  |  |  |
|  | Male | | 0 | Reference |  |  | 0 | Reference |  |
|  | Female | | -9.85 | (-12.03, -7.67) | <0.0005 |  | -7.61 | (-10.96, -4.26) | <0.0005 |
| **Change in MVPA (min/day/year)** | | | -1.55 | (-1.98, -1.12) | <0.0005^1^ |  | -2.38 | (-3.06, -1.70) | <0.0005^1^ |
| Gender | | |  |  |  |  |  |  |  |
|  | Male | | 0 | Reference |  |  | 0 | Reference |  |
|  | Female | | -0.99 | (-1.56,-0.42) | 0.001^1^ |  | -1.09 | (-2.02, -0.17) | 0.021^1^ |
| **Model 3** | | | **n=1747** |  |  |  | **n=1629** |  |  |
| **Mean MVPA at age 6 (min/day)** | | | 71.88 | (68.91, 74.85) |  |  | 69.22 | (64.93, 73.51) |  |
| Household education | | |  |  |  |  |  |  |  |
|  | Up to GCSE or equiv | | 0 | Reference |  |  | 0 | Reference |  |
|  | A level or equiv | | -2.04 | (-5.49, 1.42) |  |  | -2.41 | (-7.68, 2.86) |  |
|  | Degree or equiv | | -3.48 | (-6.78, -0.19) |  |  | -0.22 | (-5.19, 4.74) |  |
|  | Higher degree or equiv | | -6.52 | (-10.45, -2.59) | 0.010^1^ |  | -2.01 | (-7.83, 3.81) | 0.700^1^ |
| **Change in MVPA (min/day/year)** | | | -2.71 | (-3.41, -2.02) | <0.0005 |  | -2.76 | (-3.88, -1.64) | <0.0005^1^ |
| Household education | | |  |  |  |  |  |  |  |
|  | Up to GCSE or equiv | | 0 | Reference |  |  | 0 | Reference |  |
|  | A level or equiv | | 0.19 | (-0.70, 1.07) |  |  | -0.29 | (-1.75, 1.17 |  |
|  | Degree or equiv | | 0.83 | (-0.00, 1.66) |  |  | -0.43 | (-1.78, 0.92) |  |
|  | Higher degree or equiv | | 1.12 | (0.14, 2.10) | 0.050^1^ |  | -0.29 | (-1.86, 1.29) | 0.943^1^ |

All models adjusted for accelerometer wear time and seasonal variation.

^1^ Test for differences between categories.

**Table S3: Associations with change in sedentary time over time: models for all data, by gender and by household education**

|  |  | | Weekday | | |  | Weekend | | |
| --- | --- | --- | --- | --- | --- | --- | --- | --- | --- |
|  |  | | Est | 95% CI | p-value |  | Est | 95% CI | p-value |
| **Model 1** | | | **n=1924** | **n=1773** | | | |  | |
| Mean sed at age 6 (min/day) | | | 398.48 | (394.87, 402.10) |  |  | 356.72 | (352.25, 361.20) |  |
| Change in sed (min/day/year) | | | 12.90 | (12.19, 13.60) | <0.0005 |  | 13.91 | (12.96, 14.87) | <0.0005 |
| **Model 2** | | **n=1924** | | **n=1773** | | | |  | |
| **Mean sed at age 6 (min/day)** | | | 390.30 | (386.01, 394.60) |  |  | 353. 52 | (348.03, 359.02) |  |
| Gender | | |  |  |  |  |  |  |  |
|  | Male | | 0 | Reference |  |  | 0 | Reference |  |
|  | Female | | 16.68 | (11.74, 21.61) | <0.0005^1^ |  | 6.76 | (0.04, 13.48) | 0.049^1^ |
| **Change in sed (min/day/year)** | | | 12.42 | (11.47, 13.38) | <0.0005 |  | 13.99 | (12.65, 15.33) | <0.0005 |
| Gender | | |  |  |  |  |  |  |  |
|  | Male | | 0 | Reference |  |  | 0 | Reference |  |
|  | Female | | 0.63 | (-0.64, 1.89) | 0.331^1^ |  | -0.30 | (-2.13, 1.52) | 0.744^1^ |
| **Model 3** | | | **n=1747** |  |  |  | **n=1629** |  |  |
| **Mean sed at age 6 (min/day)** | | | 387.55 | (381.10, 394.01) |  |  | 354.85 | (346.35, 363.35) |  |
| Household education | | |  |  |  |  |  |  |  |
|  | Up to GCSE or equiv | | 0 | Reference |  |  | 0 | Reference |  |
|  | A level or equiv | | 10.07 | (2.45, 17.69) |  |  | 5.57 | (-4.82, 15.97) |  |
|  | Degree or equiv | | 13.90 | (6.64, 21.16) |  |  | -0.33 | (-10.13, 9.48) |  |
|  | Higher degree or equiv | | 23.74 | (15.09, 32.40) | <0.0005^1^ |  | 8.67 | (-2.83, 20.17) | 0.225^1^ |
| **Change in sed (min/day/year)** | | | 14.86 | (13.33, 16.39) | <0.0005^1^ |  | 13.15 | (10.96, 15.34) | <0.0005^1^ |
| Household education | | |  |  |  |  |  |  |  |
|  | Up to GCSE or equiv | | 0 | Reference |  |  | 0 | Reference |  |
|  | A level or equiv | | -1.70 | (-3.65, 0.26) |  |  | 0.13 | (-2.72, 2.98) |  |
|  | Degree or equiv | | -2.22 | (-4.05, -0.39) |  |  | 1.95 | (-0.68, 4.59) |  |
|  | Higher degree or equiv | | -3.70 | (-5.86, -1.53) | 0.008^1^ |  | -0.16 | (-3.23, 2.91) | 0.260^1^ |

^1^ Model adjusted for accelerometer wear time and seasonal variation.

^2^ Test for differences between categories.

**Table S4: Associations of BMI category with change in MVPA over time: full model details**

|  |  | Weekday | | |  | Weekend | | |
| --- | --- | --- | --- | --- | --- | --- | --- | --- |
|  |  | Est | 95% CI | p-value |  | Est | 95% CI | p-value |
|  | | n=1742 | n=1624 | | | |  | |
| **Mean MVPA at age 6 (min/day)** | | 75.71 | (72.62, 78.81) |  |  | 73.14 | (68.57, 77.72) |  |
| BMI category | |  |  |  |  |  |  |  |
|  | Healthy | 0 | Reference |  |  | 0 | Reference |  |
|  | Overweight | 0.99 | (-2.41, 4.38) |  |  | -0.95 | (-6.25, 4.35) |  |
|  | Obese | -0.03 | (-4.26, 4.21) | 0.846^1^ |  | -6.82 | (-13.66, 0.03) | 0.147^1^ |
| Gender | |  |  |  |  |  |  |  |
|  | Male | 0 | Reference |  |  | 0 | Reference |  |
|  | Female | -9.13 | (-11.36, -6.91) | <0.0005^1^ |  | -7.83 | (-11.25, -4.41) | <0.0005^1^ |
| Household education | |  |  |  |  |  |  |  |
|  | Up to GCSE or equiv | 0 | Reference |  |  | 0 | Reference |  |
|  | A level or equiv | -1.38 | (-4.71, 1.94) |  |  | -2.14 | (-7.34, 3.07) |  |
|  | Degree or equiv | -2.86 | (-6.03, 0.32) |  |  | 0.40 | (-4.50, 5.31) |  |
|  | Higher degree or equiv | -5.82 | (-9.60, -2.05) | 0.017^1^ |  | -1.63 | (-7.39, 4.13) | 0.648^1^ |
| **Change in MVPA (min/day/year)** | | -1.31 | (-2.07, -0.54) | 0.001 |  | -1.51 | (-2.76, -0.27) | 0.017 |
| BMI category | |  |  |  |  |  |  |  |
|  | Healthy | 0 | Reference |  |  | 0 | Reference |  |
|  | Overweight | -1.71 | (-2.65, -0.78) |  |  | -1.31 | (-2.79, 0.17) |  |
|  | Obese | -1.99 | (-3.05, -0.92) | <0.0005^1^ |  | -0.65 | (-2.42, 1.12) | 0.195^1^ |
| Gender | |  |  |  |  |  |  |  |
|  | Male | 0 | Reference |  |  | 0 | Reference |  |
|  | Female | -1.10 | (-1.67, -0.52) | <0.0005^1^ |  | -1.05 | (-2.00, -0.10) | 0.030^1^ |
| Household education | |  |  |  |  |  |  |  |
|  | Up to GCSE or equiv | 0 | Reference |  |  | 0 | Reference |  |
|  | A level or equiv | 0.13 | (-0.74, 1.00) |  |  | -0.23 | (-1.68, 1.22) |  |
|  | Degree or equiv | 0.52 | (-0.28, 1.34) |  |  | -0.78 | (-2.12, 0.57) |  |
|  | Higher degree or equiv | 0.75 | (-0.21, 1.72) | 0.332^1^ |  | -0.54 | (-2.12, 1.03) | 0.671^1^ |

^1^ Test for differences between categories.

Model adjusted for seasonality, wear time, gender, household education and age-interactions with gender and household education.

**Table S5: Associations of BMI category with change in sedentary time over time: full model details**

|  |  | Weekday | | |  | Weekend | | |
| --- | --- | --- | --- | --- | --- | --- | --- | --- |
|  |  | Est | 95% CI | p-value |  | Est | 95% CI | p-value |
|  | | n=1742 | n=1624 | | | |  | |
| **Mean sed at age 6 (min/day)** | | 383.59 | (376.67, 390.52) |  |  | 353.21 | (343.96, 362.45) |  |
| BMI category | |  |  |  |  |  |  |  |
|  | Healthy | 0 | Reference |  |  | 0 | Reference |  |
|  | Overweight | -7.90 | (-15.57, -0.22) |  |  | -3.28 | (-13.88, 7.31) |  |
|  | Obese | -11.41 | (-21.00, -1.82) | 0.014^1^ |  | -4.06 | (-17.79, 9.68) | 0.728^1^ |
| Gender | |  |  |  |  |  |  |  |
|  | Male | 0 | Reference |  |  | 0 | Reference |  |
|  | Female | 14.97 | (9.90, 20.03) |  |  | 6.15 | (-0.74, 13.05) | 0.080^1^ |
| Household education | |  |  |  |  |  |  |  |
|  | Up to GCSE or equiv | 0 | Reference |  |  | 0 | Reference |  |
|  | A level or equiv | 7.95 | (0.38, 15.51) |  |  | 4.98 | (-5.51, 15.47) |  |
|  | Degree or equiv | 11.84 | (4.63, 19.05) |  |  | -1.23 | (-11.12, 8.66) |  |
|  | Higher degree or equiv | 21.74 | (13.16, 30.32) | <0.0005^1^ |  | 7.35 | (-4.27, 18.96) | 0.271^1^ |
| **Change in sed (min/day/year)** | | 13.20 | (11.49, 14.91) | <0.0005 |  | 12.61 | (10.15, 15.08) | <0.0005 |
| BMI category | |  |  |  |  |  |  |  |
|  | Healthy | 0 | Reference |  |  | 0 | Reference |  |
|  | Overweight | 2.90 | (0.80, 5.01) |  |  | 1.91 | (-1.04, 4.86) |  |
|  | Obese | 3.05 | (0.65, 5.45) | 0.002^1^ |  | 1.50 | (-2.01, 5.01) | 0.352^1^ |
| Gender | |  |  |  |  |  |  |  |
|  | Male | 0 | Reference |  |  | 0 | Reference |  |
|  | Female | 1.07 | (-0.22, 2.37) | 0.104^1^ |  | -0.17 | (-2.05, 1.70) | 0.856^1^ |
| Household education | |  |  |  |  |  |  |  |
|  | Up to GCSE or equiv | 0 | Reference |  |  | 0 | Reference |  |
|  | A level or equiv | -1.36 | (-3.32, 0.59) |  |  | 0.24 | (-2.64, 3.11) |  |
|  | Degree or equiv | -1.72 | (-3.56, 0.11) |  |  | 2.24 | (-0.42, 4.91) |  |
|  | Higher degree or equiv | -3.25 | (-5.42, -1.07) | 0.033^1^ |  | 0.18 | (-2.93, 3.29) | 0.208^1^ |

^1^ Test for differences between categories.

Model adjusted for seasonality, wear time, gender, household education and age-interactions with gender and household education.

**Table S6: Change in MVPA and sedentary time over time by BMI category for 3 valid weekdays and 2 valid weekends: sensitivity analysis**

|  |  | Weekday | | |  | Weekend | | | |
| --- | --- | --- | --- | --- | --- | --- | --- | --- | --- |
|  |  | Est | 95% CI | p-value |  | Est | 95% CI | | p-value |
| **MVPA** | | n=1461 |  |  |  | n=1213 |  | |  |
| **Mean MVPA at age 6 (min/day)** | | 75.64 | (72.17, 79.10) |  |  | 73.06 | (67.74, 78.39) | |  |
| BMI category | |  |  |  |  |  |  | |  |
|  | Healthy | 0 | Reference |  |  | 0 | Reference | |  |
|  | Overweight | 1.40 | (-2.39, 5.18) |  |  | -0.12 | (-6.03, 5.80) | |  |
|  | Obese | -0.81 | (-5.54, 3.92) | 0.702^1^ |  | -4.00 | (-12.58, 4.59) | | 0.659^1^ |
| **Change in MVPA (min/day/year)** | | -1.14 | (-2.01, -0.27) | 0.010 |  | -1.37 | (-2.83, 0.09) | | 0.066 |
| BMI category | |  |  |  |  |  | |  |  |
|  | Healthy | 0 | Reference |  |  | 0 | Reference | |  |
|  | Overweight | -1.42 | (-2.46, -0.37) |  |  | -1.66 | (-3.32, -0.01) | |  |
|  | Obese | -1.84 | (-3.04, -0.64) | 0.001^1^ |  | -1.49 | (-3.71, 0.73) | | 0.079^1^ |
| **Sedentary Time** | | n=1461 |  |  |  | n=1213 |  | |  |
| **Mean sed time at age 6 (min/day)** | | 387.64 | (379.91, 395.36) |  |  | 362.91 | (351.94, 373.89) | |  |
| BMI category | |  |  |  |  |  |  | |  |
|  | Healthy | 0 | Reference |  |  | 0 | Reference | |  |
|  | Overweight | -7.94 | (-16.53, 0.65) |  |  | -3.72 | (-15.86, 8.43) | |  |
|  | Obese | -6.60 | (-17.35, 4.15) | 0.120^1^ |  | -1.05 | (-18.69, 16.59) | | 0.834^1^ |
| **Change in sed time (min/day/year)** | | 12.74 | (10.77, 14.71) | <0.0005 |  | 12.13 | (9.15, 15.11) | | <0.0005 |
| BMI category | |  |  |  |  |  | |  |  |
|  | Healthy | 0 | Reference |  |  | 0 | Reference | |  |
|  | Overweight | 2.88 | (0.51, 5.25) |  |  | 2.06 | (-1.32, 5.45) | |  |
|  | Obese | 1.64 | (-1.09, 4.36) | 0.039^1^ |  | 0.77 | (-3.76, 5.31) | | 0.480^1^ |

^1^ Test for differences between BMI categories.

Model adjusted for seasonality, wear time, gender, household education and age-interactions with gender and household education.
